# Cell Wall Composition and Biomass Recalcitrance Differences Within a Genotypically Diverse Set of *Brachypodium distachyon* Inbred Lines

**DOI:** 10.3389/fpls.2016.00708

**Published:** 2016-05-26

**Authors:** Cynthia L. Cass, Anastasiya A. Lavell, Nicholas Santoro, Cliff E. Foster, Steven D. Karlen, Rebecca A. Smith, John Ralph, David F. Garvin, John C. Sedbrook

**Affiliations:** ^1^School of Biological Sciences, Illinois State University, NormalIL, USA; ^2^U.S. Department of Energy Great Lakes Bioenergy Research Center, University of Wisconsin-Madison, MadisonWI, USA; ^3^Department of Agronomy and Plant Genetics, University of Minnesota, St. PaulMN, USA; ^4^Plant Science Research Unit, United States Department of Agriculture, Agricultural Research Service, St. PaulMN, USA; ^5^U.S. Department of Energy Great Lakes Bioenergy Research Center, Michigan State University, East LansingMI, USA; ^6^Department of Biochemistry, University of Wisconsin-Madison, MadisonWI, USA

**Keywords:** bioenergy, grass, polysaccharide, hemicellulose, lignin, digestibility, vernalization, Pooideae

## Abstract

*Brachypodium distachyon* (*Brachypodium*) has emerged as a useful model system for studying traits unique to graminaceous species including bioenergy crop grasses owing to its amenability to laboratory experimentation and the availability of extensive genetic and germplasm resources. Considerable natural variation has been uncovered for a variety of traits including flowering time, vernalization responsiveness, and above-ground growth characteristics. However, cell wall composition differences remain underexplored. Therefore, we assessed cell wall-related traits relevant to biomass conversion to biofuels in seven *Brachypodium* inbred lines that were chosen based on their high level of genotypic diversity as well as available genome sequences and recombinant inbred line (RIL) populations. Senesced stems plus leaf sheaths from these lines exhibited significant differences in acetyl bromide soluble lignin (ABSL), cell wall polysaccharide-derived sugars, hydroxycinnamates content, and syringyl:guaiacyl:*p*-hydroxyphenyl (S:G:H) lignin ratios. Free glucose, sucrose, and starch content also differed significantly in senesced stems, as did the amounts of sugars released from cell wall polysaccharides (digestibility) upon exposure to a panel of thermochemical pretreatments followed by hydrolytic enzymatic digestion. Correlations were identified between inbred line lignin compositions and plant growth characteristics such as biomass accumulation and heading date (HD), and between amounts of cell wall polysaccharides and biomass digestibility. Finally, stem cell wall *p*-coumarate and ferulate contents and free-sugars content changed significantly with increased duration of vernalization for some inbred lines. Taken together, these results show that *Brachypodium* displays substantial phenotypic variation with respect to cell wall composition and biomass digestibility, with some compositional differences correlating with growth characteristics. Moreover, besides influencing HD and biomass accumulation, vernalization was found to affect cell wall composition and free sugars accumulation in some *Brachypodium* inbred lines, suggesting genetic differences in how vernalization affects carbon flux to polysaccharides. The availability of related RIL populations will allow for the genetic and molecular dissection of this natural variation, the knowledge of which may inform ways to genetically improve bioenergy crop grasses.

## Introduction

*Brachypodium distachyon* (*Brachypodium*) is an annual grass species native to southern Europe, northern Africa, the Middle East, and southwestern Asia. As a member of the grass subfamily Pooideae, *Brachypodium* is related to *Triticum aestivum* (wheat) and other temperate cereals as well as most of the forage grass species, making it a useful model for a wide range of biological aspects of cool season grass biology (Draper et al., 2001; Mur et al., 2011; Barrero et al., 2012; Cui et al., 2012; Lee M.Y. et al., 2012; Figueroa et al., 2015; Zhong et al., 2015). The cell wall composition, growth architecture, and flowering time regulation of *Brachypodium* are similar to those of other grass species, making it a useful model for biomass improvement of dedicated bioenergy grass species such as switchgrass and Miscanthus (Gomez et al., 2008; Vogel, 2008; Lee S.J. et al., 2012; Rancour et al., 2012).

*Brachypodium* has a diploid genome that is one of the smallest and least repetitive of any grass species, which allowed for the rapid generation of a high-quality reference genome sequence for inbred line Bd21 (International Brachypodium Initiative, 2010), followed by additional genome sequences from several genotypically diverse *Brachypodium* genotypes (Vogel et al., 2009; Gordon et al., 2014). The genome sequences and germplasm resources for *Brachypodium* coupled with its simple growth requirements and inbred nature, make this species attractive for studies of the molecular basis of natural variation.

Multiple studies have shown that *Brachypodium* exhibits extensive natural variation with respect to photoperiod responsiveness, vernalization requirements, and flowering time, with accessions broadly falling into winter and spring annual types (Schwartz et al., 2010; Ream et al., 2014; Woods et al., 2014a,b). Luo et al. (2011) surveyed 57 natural populations of *Brachypodium* for drought tolerance, and found significant phenotypic diversity based on principal component (PC) analyses of chlorophyll fluorescence and leaf water content under drought stress. Tyler et al. (2014) also identified considerable phenotypic diversity within a large collection of inbred *Brachypodium* lines, focusing on bioenergy-relevant traits including plant height, growth habit, stem density, and cell wall composition as inferred by near infrared spectroscopy (NIR) and comprehensive microarray polymer profiling (CoMPP).

Although the Tyler et al. (2014) study identified significant *Brachypodium* natural variation with respect to hemicellulose and pectin compositions, the analyses were semi-quantitative and did not explore lignin composition or biomass recalcitrance, two traits centrally important to developing bioenergy crops for conversion to liquid biofuels. Rancour et al. (2012) performed a detailed quantitative analysis of *Brachypodium* cell wall composition including that of lignin in different tissue types throughout development, but did not assess phenotypic diversity or biomass recalcitrance. Therefore, for this study we chose seven inbred lines that were previously found to have a high level of genotypic diversity (Vogel et al., 2009), and phenotypically assessed all of the major secondary cell wall components as well as possible relationships to biomass recalcitrance. The chosen lines are particularly useful in that their genome sequences are publicly available, as are recombinant inbred line (RIL) populations for crosses between many of them (Gordon et al., 2014; Garvin, 2015). These findings and resources will facilitate and accelerate discovery of the underlying mechanisms controlling yield, biomass composition, and recalcitrance to conversion to biofuels.

## Materials and Methods

### Materials

Used in this study were the *Brachypodium* inbred lines Bd1-1, Bd2-3, Bd3-1, Bd18-1, Bd21, Bd21-3, and Bd30-1. These lines were developed by varying generations of single seed descent inbreeding (Vogel et al., 2006; Garvin et al., 2008; Vogel and Hill, 2008; Garvin, 2015). For each experiment, pots containing plants for each line were grown in an intermixed manner to limit variation due to environmental effects. Experiments were repeated at least three times, producing similar results.

### Growth Conditions

For biomass trait and cell wall analyses of the inbred lines, surface-sterilized seeds were germinated on half-strength Murashige and Skoog (MS) no sucrose plates [2.3 g L^-1^ MS salts, 15 g L^-1^ BD granulated agar, 0.6 g L^-1^ 2-(4-morpholino)-ethane sulfonic acid (MES) pH 5.7], stratified for 3 days at 6°C in the dark, then moved to a Percival growth chamber with a 16 h light: 8 h dark photoperiod, 22°C, 109 μmol m^-2^ s^-1^ fluorescent light for 5–7 days before planting. Sixty randomly chosen seedlings for each line were transferred to 10 cm pots, at a density of five plants per pot, containing a 50:50 mix of SunGro Rediearth and MetroMix 510 soil supplemented with the insecticide Marathon (29.1 mg per 10 cm pot) and the fungicide Bayleton (5.7 mg per 10 cm pot). Pots were randomly placed in EGC growth chambers with a 20 h light: 4 h dark photoperiod (mixture of fluorescent and incandescent light), 22°C, 50% humidity, and approximately 225–350 μmol m^-2^ s^-1^ light intensity. Winter habit lines Bd1-1 and Bd18-1 were grown for 4 weeks in the growth chamber, then vernalized for 7 weeks at 6°C with 24 h light, and moved back into the growth chamber. To have all of the inbred lines mature together in a growth chamber, the five spring habit lines (Bd2-3, Bd21, Bd21-3, Bd3-1, and Bd30-1) were germinated 2 weeks prior to removing Bd1-1 and Bd18-1 from the vernalization treatment. Plants were fertilized approximately 2 weeks after planting with MiracleGro 20-20-20 (1/2 teaspoon per gallon).

To assess the effects of vernalization on the spring habit lines Bd2-3, Bd21, and Bd30-1, seeds were planted into 7.6 cm square pots (eight seeds per pot), placed just below the surface of SB500 soil saturated with Plantex fertilizer solution (2 g fertilizer L^-1^). At planting, pots were covered with plastic wrap and placed in 4°C deli cooler to vernalize. The first planted set remained in the cooler for 28 days, the second set for 21 days, the third set for 14 days, the fourth set for 7 days, and the fifth planted set was not vernalized. The day that the pots of unvernalized plants were planted, the other pots were taken out of the deli cooler and all pots moved together to a Conviron growth chamber. The growth chamber conditions were 22°C with a 20 h light: 4 h dark photoperiod. Lights were run at half strength intensity, which typically ranges from 100 to 150 μmol m^-2^ s^-1^ depending on bulb age. The pots were divided into three replicate trays, with each tray containing a pot for each line that had been vernalized for each of the different lengths of time. Pots within each tray were randomized. After seedling emergence, pots were thinned to four healthy plants. All plants were watered with tap water as needed, and allowed to mature, senesce, and dry completely before harvesting.

### Biomass Trait Data Collection

Growth habit observations for the height from the soil to the awn of the tallest culm, above-ground mass, number of primary and secondary culms, and heading date (HD) were recorded individually for each plant. Pot HD was defined as the number of days until three out of the five plants in a given pot had visible spikes. To ensure that growth habit observations would be consistent with subsequent cell wall analyses that would require significant pooling of plant material, individual plant values were averaged by pot to generate 12 biological replicate means per trait for each inbred line. For cell wall compositional analyses, 12 biological tissue replicates were generated by collecting senesced stem plus leaf sheath tissue, with rachis and leaf blades removed, from the top two internodes of the three tallest culms from each plant individually, and then pooling tissue by pot, within each inbred line. For the vernalization studies on spring types Bd2-3, Bd21, and Bd30-1, HD was recorded for each pot replicate as the date on which the start of heading (spike emergence) occurred on at least two of the four plants in a pot. All plants were staked and tied up as needed, then allowed to mature, senesce, and dry before harvesting. Trait means and their standard errors were obtained using the pot averages.

### Cell Wall Polysaccharide Compositional Analysis

Senesced stem plus leaf sheath tissue from 12 biological replicates per inbred line was ball-milled, water and solvent extracted, and then destarched to generate alcohol-insoluble residue (AIR) cell wall material. Destarching was performed in a pH 5.0-buffered solution of 1.2 μg/mL amylase (Sigma–Aldrich) and 12.5 U/mL pullulanase (Sigma–Aldrich) at 37°C overnight, as previously described (Foster et al., 2010). Matrix polysaccharide composition and crystalline cellulose content was determined as described in Albersheim et al. (1967) and Foster et al. (2010). Briefly, polysaccharide composition was determined by GC-MS separation on the alditol acetates resulting from a 2 M trifluoroacetic acid (TFA) hydrolysis, reduction (NaBH_4_) of the neutral monosaccharides present the hydrolysate supernatant, and subsequent acetylation. Crystalline cellulose content was determined by treating the cell wall material not hydrolyzed by TFA with Updegraff reagent, an acid mix that results in further stripping of hemicelluloses and amorphous glucan from the cell wall material (Updegraff, 1969). The residual crystalline cellulose was hydrolyzed with sulfuric acid (Selvendran and O’Neill, 1987) and the resulting monosaccharide (glucose) was quantified using a colorimetric anthrone assay.

### Lignin Content

Extractive-free AIR (above) was solubilized with acetyl bromide (Fukushima and Hatfield, 2001) and lignin content determined by Beer’s Law from the UV absorbance at 280 nm using a corn stover molar extinction coefficient of 17.75 g^-1^ L cm^-1^ (Fukushima and Hatfield, 2004) and a 1 cm pathlength.

### Lignin Composition

Lignin composition from twelve biological replicates per inbred line was determined by thioacidolysis based on the original method (Lapierre, 2010). Briefly, the thioacidolysis reagent (2.5% (v/v) boron trifluoride diethyletherate, 10% (v/v) ethanethiol in freshly distilled dioxane) was spiked with 4,4′-ethylidenebisphenol (1 mg/mL in dioxane) as an internal standard. Thioacidolysis monomers were extracted after 4 h at 100°C, then silylated with *N,O*-bis(trimethylsilyl)trifluoroacetamide and pyridine, and quantified by GC/MS [Agilent GC/MS (6890 GC/5975B MS) fitted with a Supelco SLB-5MS column (30 mm × 0.25 mm × 0.25 μm film)] using synthetic thioacidolysis monomers as standards. The linear range and response factor (RF) for the synthetic monomers were: for S, 25–300 μg, *r*^2^ = 0.998, RF (ion 299 vs. 343 of Bisphenol E) = 2.16; for G, 25–300 μg, *r*^2^ = 0.998, RF (ion 269 vs. 343 of Bisphenol E) = 2.15; and for H, 2.5–50 μg, *r*^2^ = 0.998, RF (ion 239 vs. 343 of Bisphenol E) = 2.11.

### Hydroxycinnamates Determination

Extractive-free culm and leaf sheath cell walls from three randomly selected biological replicate tissue pools for each inbred line were subjected to saponification to release the *p*CA and FA. Subsequent quantification was performed following the procedures reported by Ralph et al. (1994).

### Pretreatment, Saccharification, and Sugar Quantification

Culm plus leaf sheath biomass from 12 biological replicates per inbred line was subjected to one of a suite of pretreatments (grinding alone, or grinding with either (a) 90°C hot water, (b) 6.25 mM NaOH, (c) 62.5 mM NaOH, or (d) 4% (v/v) H_2_SO_4_), and subjected to partial hydrolytic enzyme saccharification by adding a pH 4.5-buffered solution of Accellerase 1000 (Genencor) at 50°C for 20 h. Colorimetric glucose and pentose quantifications were performed using either the glucose oxidase/peroxidase (GOPOD) method (*K*-GLUC, Megazyme) for glucose amounts, or the *p*-bromoaniline in thiourea method (Deschatelets and Yu, 1986) for pentose amounts as previously described (Santoro et al., 2010; Cass et al., 2015).

### Determination of Free Glucose, Sucrose, and Starch

The free glucose, sucrose, and starch contents of culm plus leaf sheath biomass samples from 12 biological replicates per inbred line was determined as previously described (Santoro et al., 2010; Cass et al., 2015). In short, the same GOPOD method (*K*-GLUC, Megazyme) was employed to quantify free glucose washed with distilled water from pulverized biomass as well as glucose released from sucrose using an invertase cocktail and glucose released from starch using amyloglucosidase and α-amylase.

### Statistical Analyses

For experiments involving all seven inbred lines, twelve biological replicates were analyzed (*n* = 12, where *n* was the number of pots for each line; each 10 cm pot contained five plants whose growth measurements were averaged and senesced stem tissue pooled for cell wall analyses). A *M*ultivariate *An*alysis *o*f *Va*riance (MANOVA) was performed to determine the effect of line across and within groups for dependent variable means observed for growth traits and cell wall composition using PROC GLM in SAS v. 9.3 (SAS Institute Inc., Cary, NC, USA; *p* < 0.05). Dependent variables for growth traits included HD (not including time of vernalization), plant height, and log_10_(plant mass) and log_10_(number of culms). Dependent variables for cell wall composition included: acetyl bromide soluble lignin (ABSL) amount; lignin syringyl (S), guaiacyl (G), and *p*-hydroxyphenyl (H) monomer amounts released by thioacidolysis; neutral sugars xylose, arabinose, galactose, rhamnose, fucose, and glucose; crystalline cellulose; and log_10_(mannose) amounts. Means for dependent variables with significant individual *An*alysis *o*f *Va*riance (ANOVA) results after applying a Bonferroni correction (α′ = 0.003) were separated by Tukey’s Studentized Range (HSD) test to stringently control for Type I error. Correlations between growth trait and cell wall means were calculated with PROC CORR with a Bonferroni correction (α′ = 0.0003) applied for significance cut off. Log_10_ transformations were carried out in instances where the data were not normally distributed so as to meet the assumptions for ANOVA.

*An*alysis *o*f *Va*riance was performed to compare the main effect of line across and within groups for both *p*-coumarate and ferulate amounts, or the derived variable, log_10_(S:G ratio) using PROC GLM with a Bonferroni correction (α′ = 0.02). Means were separated as above.

*An*alysis *o*f *Va*riance, with a Bonferroni correction, was performed to determine the effect of line across and within groups for glucose and pentose release following a set of thermochemical pretreatments using PROC GLM (α′ = 0.005). Means for significant results were treated as above.

*P*rincipal *C*omponent *A*nalysis (PCA) was performed on HD, cell wall composition (excluding *p*-coumarate, ferulate, and non-cell wall components) means along with a derived variable describing the overall growth habit for each of the inbred lines, biomass index (BI = height × mass/culm) means using PROC FACTOR. The principal axis method was used to extract the components, followed by a varimax (orthogonal) rotation. Significance cut off for factor loading was 0.4. The correlations between PC and digestibility were analyzed by PROC CORR.

Statistically significant differences related to the effects of vernalization on growth traits, cell wall composition and biomass digestibility for spring inbred lines Bd21, Bd30-1, and Bd2-3 were determined by performing PROC REG (α′ = 0.002; *N* = 9; *n* = 3 where *n* was the number of pots for each line. Each 7.6 cm pot contained four plants whose growth characteristics were averaged and senesced stem tissue pooled for cell wall analyses).

## Results

Recent studies have shown that *Brachypodium* germplasm collected throughout its growth range is genotypically highly diverse (Vogel et al., 2009; Gordon et al., 2014). We set out to determine if genotypically diverse germplasm possessed comparable phenotypic natural variation with respect to cell wall composition and biomass recalcitrance, which is relevant to exploring ways to improve grasses as feedstocks for biofuels generation. We analyzed seven inbred lines, namely Bd21, Bd21-3, Bd3-1, Bd2-3, Bd30-1, Bd1-1, and Bd18-1. These lines were chosen because they had been inbred by single seed descent (Vogel et al., 2006; Garvin et al., 2008; Vogel and Hill, 2008; Garvin, 2015) thereby increasing homozygosity beyond the very high level that is already present due to the high level of inbreeding that occurs naturally (Vogel et al., 2009). Moreover, these lines are genotypically highly diverse based on the phylogenetic analysis published in Vogel et al. (2009). Their genome sequences are publically available and many of the lines have been crossed with each other to generate RIL populations, which will facilitate genetic interrogation of the observed natural variation (Garvin et al., 2008; Garvin, 2015).

Previous studies determined that inbred lines Bd21, Bd21-3, Bd3-1, Bd2-3, and Bd30-1 are spring annuals (do not require vernalization to flower) whereas Bd1-1 and Bd18-1 are winter annuals, requiring up to 6 weeks of cold treatment to flower (Schwartz et al., 2010; Tyler et al., 2014). Inbred line Bd21 was used in generating the *Brachypodium* reference genome (International Brachypodium Initiative, 2010), whereas inbred line Bd21-3 has been used in generating various community resources including EST and sequence-indexed mutant collections (Vogel et al., 2006; Bragg et al., 2012).

### Analysis of Phenotypic Variation

A MANOVA analysis was performed to generate standard canonical coefficients that described the observed variation for the effect of line on growth trait and cell wall composition biological replicate means (*p* ≤ 0.05, 7 lines; *N* = 84, *n* = 12). With only seven inbred lines in the set, we did not differentiate between spring and winter habit lines. Due to the lack of a high-throughput method to determine cell wall *p*-coumarate (*p*CA) and ferulate (FA) amounts, only three biological replicate means per inbred line were generated for those analyses. To avoid the loss of statistical power in the analyses of data with 12 biological replicates per line, the variation of *p*CA and FA across and within lines was determined separately.

The MANOVA analysis of growth trait and cell wall composition identified a significant multivariate effect across and within groups for inbred lines [Pillai’s trace = 4.80, *F*(114,384) = 13.43, *p* < 0.0001]. Since the multivariate analysis was significant, means for dependent variables with significant individual ANOVA results, after applying a Bonferroni correction (α′ = 0.003), were separated by Tukey’s Studentized Range (HSD) test (*p* ≤ 0.05). The results are discussed in the context of growth habit and cell wall composition in the following sections.

### Growth Habit

**FIGURE 1:**
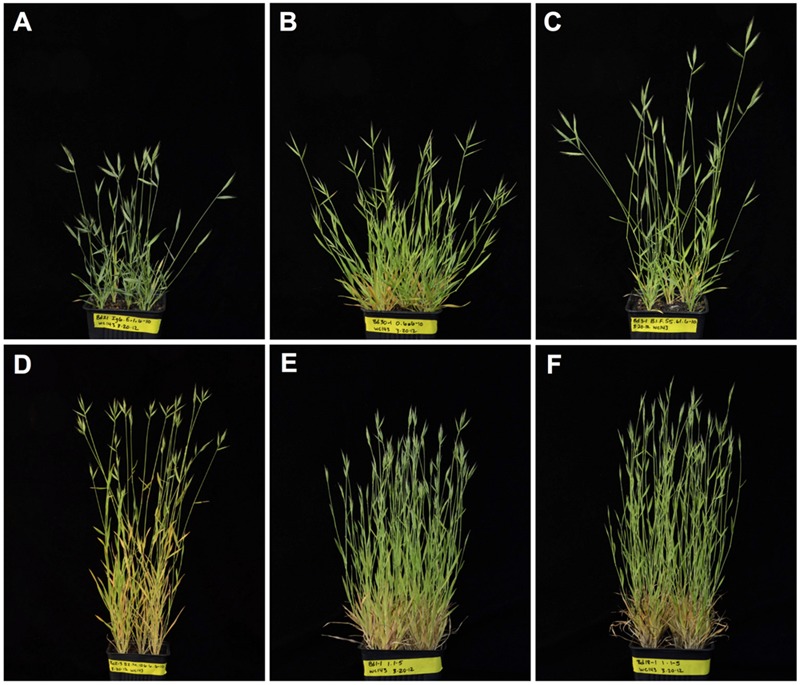
**Growth habits of *Brachypodium distachyon* inbred lines.** Representative images of non-vernalized plants from lines **(A)** Bd21, **(B)** Bd30-1, **(C)** Bd3-1 (pictures taken 44 days after planting), and **(D)** Bd2-3 (picture taken 52 days after planting), as well as vernalized plants from lines **(E)** Bd1-1 and **(F)** Bd18-1 (pictures are of 115 day-old plants, 37 days after the vernalization period). Bd21-3 plants, which are not shown, looked essentially identical to Bd21 plants. Plants were grown at a density of five plants per 10 cm pot under a 20:4 h light:dark photoperiod.

**FIGURE 2:**
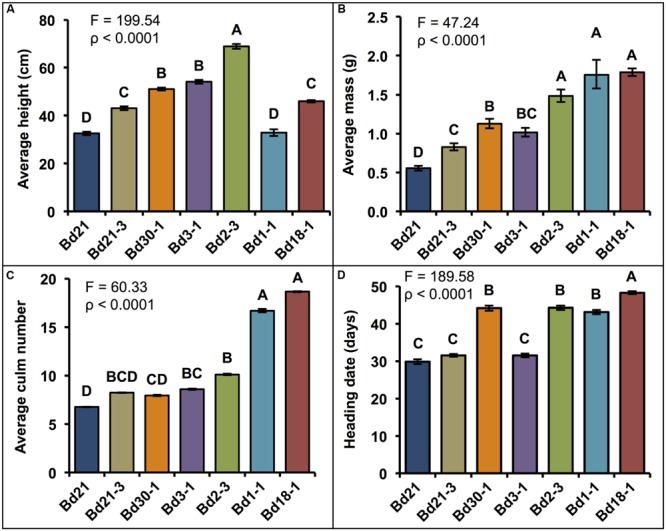
**Quantification of growth habits.** Means ± SEM for **(A)** height of each plant’s tallest culm, **(B)** aboveground dry mass (Log_10_ backtransformed means ± approximate SEM), **(C)** culm number per plant (Log_10_ backtransformed means ± approximate SEM), and **(D)** heading date (HD). Bd1-1 and Bd18-1 were vernalized 7 weeks to induce flowering (days of vernalization not included in HD). Different letters over the means indicate significant differences, α′ = 0.003, *N* = 84, *n* = 12 where *n* is the number of pots for each line (each pot contained five plants that were measured together to generate a mean).

The phenotypic differences described in this and the following sections were reproducible in at least three separate plantings. Representative images of the growth habits of plants from six of the seven inbred lines are shown in **Figure 1**. Consistent with findings from other studies (Schwartz et al., 2010; Ream et al., 2014; Tyler et al., 2014; Woods et al., 2014a,b), significant differences in the average height, mass, and number of culms were observed between inbred lines (**Figures 2A–C**). There was a trend for taller plants to have greater mass, although Bd3-1 had less mass than expected for its height, and the two winter habit lines Bd1-1 and Bd18-1 had significantly greater mass than expected for their average heights (**Figure 2B**). The latter observation may be at least partially explained by the significantly higher number of culms the winter habit lines produced compared to the spring habit lines (**Figure 2C**).

Differences were also observed in HD, which is defined as the number of days until three out of the five plants in a given pot had visible spikes (**Figure 2D**). Comparing the spring lines, which were not vernalized, Bd21, Bd21-3, and Bd3-1 plants headed 30–32 days after planting (DAP) whereas Bd30-1 and Bd2-3 headed 44 DAP, about 2 weeks later. As for the winter lines, Bd1-1 and Bd18-1 headed 43 and 48 DAP (not including the 7 weeks vernalization period), respectively.

### Lignin

**FIGURE 3:**
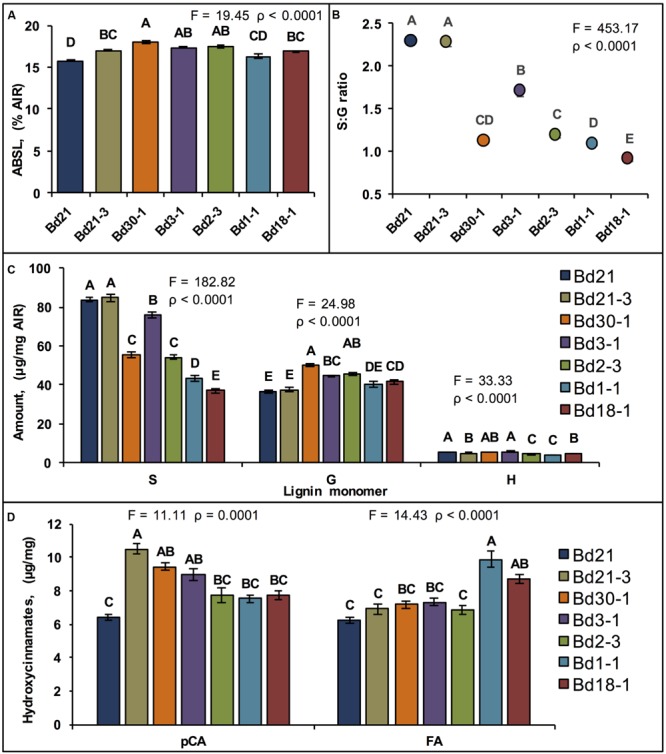
**Quantification of cell wall components.** Means ± SEM for stem plus leaf sheath cell wall **(A)** acetyl bromide soluble lignin (ABSL), **(B)** Syringyl:Guaiacyl lignin units ratio (S:G ratio, Log_10_ backtransformed means ± approximate SEM), **(C)** thioacidolysis lignin units composition, and **(D)** *p*-coumaric acid (*p*CA, left) and ferulic acid (FA, right) amounts are shown. Different letters over the means indicate significant differences, for **(A–C)**, α′ = 0.003, *N* = 84, *n* = 12 where *n* is the number of pots for each line (each pot contained five plants whose senesced stem tissue was pooled constituting a sample); for **(D)**, *N* = 6, *n* = 3, α′ = 0.02.

Lignin quantitation, performed on senesced stem plus leaf sheath biomass from the seven inbred lines using the acetyl bromide method revealed average ABSL levels ranging from 15.8% AIR for Bd21 to 18.0% AIR for Bd30-1, which was a 13% difference (**Figure 3A**). In contrast to these modest yet significant lignin quantity differences, lignin syringyl (S), guaiacyl (G), and *p*-hydroxyphenyl (H) unit compositions, as determined by the thioacidolysis method (Lapierre, 2010), varied dramatically between inbred lines (**Figures 3B,C**). S unit amounts were up to 78% different between the two least similar lines. G unit amounts were as much as 30% different (**Figure 3C**). S:G ratios ranged from a low of 0.89 for Bd18-1 to a high of 2.28 for Bd21, a greater than 2.5-fold difference (**Figure 3B**).

The relative amounts of both *p*-coumaric acid (*p*CA) and ferulic acid (FA), *p*-hydroxycinnamates that acylate lignin and hemicelluloses, were quantified upon release from solvent-extracted pulverized senesced stems plus leaf sheaths after treatment with 2 M sodium hydroxide (NaOH). Mean *p*CA amounts ranged from 6.4 mg/g AIR for Bd21 to 10.5 mg/g for Bd21-3, which was a 49% difference (**Figure 3D**). Similarly, FA amounts varied by as much as 46%. However, the relative line-to-line differences in FA content did not mirror the relative line-to-line differences in *p*CA content (**Figure 3D**).

### Cell Wall Neutral Sugar Composition

Cell wall neutral sugar composition was determined from the alditol acetates resulting from TFA hydrolysis, reduction, and acetylation. Under our controlled growth conditions, sugars attributable primarily to cell wall hemicelluloses varied significantly between inbred lines (**Table 1**). Xylose and arabinose amounts were up to 16 and 25% different between lines, respectively.

**Table 1:** Neutral sugar content of *Brachypodium distachyon* stem plus leaf sheath cell walls.

Line	Xylose	Arabinose	Fucose	Mannose	Galactose	Rhamnose	Glucose	Crystalline cellulose
Bd21	240.8 ± 3.3^b^	31.1 ± 0.6^d^	0.14 ± 0.02^a^	0.32 ± 0.01	3.8 ± 0.1^d^	0.53 ± 0.02^ab^	33.4 ± 0.9	443.6 ± 5.6^a^
Bd21-3	250.5 ± 3.4^b^	33.3 ± 0.5^cd^	0.06 ± 0.03^ab^	0.33 ± 0.01	4.7 ± 0.1^b^	0.57 ± 0.03^ab^	34.8 ± 0.9	416.0 ± 5.9^b^
Bd30-1	249.4 ± 3.1^b^	31.6 ± 0.5^d^	0.13 ± 0.02^a^	0.29 ± 0.01	4.1 ± 0.1^cd^	0.50 ± 0.02^b^	32.9 ± 0.8	414.0 ± 5.3^b^
Bd3-1	254.8 ± 3.6^b^	33.2 ± 0.6^cd^	0.01 ± 0.03^b^	0.33 ± 0.02	4.4 ± 0.2^bc^	0.47 ± 0.03^b^	32.8 ± 0.9	405.0 ± 6.2^b^
Bd2-3	272.7 ± 3.3^a^	35.0 ± 0.5^bc^	0.09 ± 0.02^ab^	0.33 ± 0.01	4.8 ± 0.1^b^	0.52 ± 0.02^ab^	32.3 ± 0.9	413.4 ± 5.6^b^
Bd1-1	282.0 ± 3.4^a^	35.8 ± 0.5^b^	0.08 ± 0.03^ab^	0.33 ± 0.01	4.7 ± 0.1^b^	0.54 ± 0.03^ab^	32.7 ± 0.9	416.7 ± 5.9^b^
Bd18-1	282.1 ± 3.8^a^	39.9 ± 0.6^a^	0.05 ± 0.03^ab^	0.33 ± 0.02	6.4 ± 0.2^a^	0.62 ± 0.03^a^	31.9 ± 1.0	399.6 ± 6.5^b^
*F* ratio	24.42	29.18	3.56	1.64	32.57	3.51	1.09	5.71
ρ	<0.0001	<0.0001	0.0042	0.1505	<0.0001	0.0046	0.3786	<0.0001

*Cell wall sugar LS means ± SEM in μg mg^-1^ AIR (Log10 backtransformed LS means ± approximate SEM for mannose) are shown. Different letters within each column indicate significant differences; α′ = 0.003.*

The amounts of galactose and rhamnose, which are found in pectins, arabinogalactan proteins (AGPs), galactans, and arabinogalactans (AGs; Carpita, 1996; Seifert and Roberts, 2007), also varied significantly by as much as 51 and 28%, respectively (**Table 1**). Fucose amounts (perhaps originating mostly from AGPs and AGs, although this remains to be determined) varied by as much as 14-fold, while conversely, mannose amounts were statistically indistinguishable between the lines (**Table 1**). Glucose originating primarily from amorphous cellulose and mixed-linkage glucan (MLG) varied relatively little between the lines. In contrast, average crystalline cellulose content was significantly higher in Bd21 senesced stems at 443.6 mg/g compared to any of the other six inbred lines, the lowest of which was 399.6 mg/g in Bd18-1, a 10% difference (**Table 1**).

In terms of total non-cellulosic cell wall sugars, the greatest differences were between Bd21 and Bd18-1 (**Table 1**). For example, galactose, arabinose, and xylose comprised 0.5, 4.1, and 31.9% of Bd21 total cell wall neutral sugar amounts, whereas Bd18-1 had significantly greater amounts at 0.8, 5.2, and 37.1% of total cell wall neutral sugars.

### Free Sugars and Starch

Striking differences were observed in the amounts of free glucose and sucrose present in senesced stems plus leaf sheaths biomass among the inbred lines (**Table 2**). At the top of the range, Bd18-1 had 4.3 and 2.5% free glucose and sucrose, respectively, and Bd2-3 had 4.0 and 2.8% free glucose and sucrose. In contrast, Bd21 averaged only 0.4% free glucose and 0.4% sucrose content under our growth conditions, respectively, which was 11 and 6-fold lower than that measured in Bd18-1. Bd2-3 and Bd21 senesced stems plus leaf sheaths had the greatest and least amounts of starch at 1.3 and 0.5%, a 2.6-fold difference (**Table 2**).

**Table 2:** Free glucose, sucrose, and starch content of *B. distachyon* stem plus leaf sheath senesced biomass.

Line	Free glucose	Sucrose	Starch
Bd21	0.4 ± 0.02^e^	0.4 ± 0.02^c^	0.5 ± 0.01^d^
Bd21-3	0.6 ± 0.04^de^	0.5 ± 0.04^c^	0.7 ± 0.04^cd^
Bd30-1	1.1 ± 0.14^d^	0.7 ± 0.07^c^	0.8 ± 0.07^bc^
Bd3-1	1.9 ± 0.15^c^	1.3 ± 0.11^b^	0.8 ± 0.07^bc^
Bd2-3	4.0 ± 0.23^a^	2.8 ± 0.17^a^	1.3 ± 0.1^a^
Bd1-1	2.7 ± 0.17^b^	1.6 ± 0.11^b^	0.9 ± 0.05^bc^
Bd18-1	4.3 ± 0.23^a^	2.5 ± 0.17^a^	1.0 ± 0.09^b^
*F* ratio	99.93	64.43	16.34
Prob.	ρ < 0.0001	ρ < 0.0001	ρ < 0.0001

*Values represent % yield dry weight ± SEM. Different letters indicate significant differences; α′ = 0.003.*

### Digestibility

To assess biomass digestibility, we employed a previously published high-throughput digestibility platform (HTDP, Santoro et al., 2010), utilizing five different pretreatments followed by hydrolytic enzyme saccharification and colorimetric quantitation of glucose and pentose sugars. Pretreatments included grinding alone as well as grinding plus hot water, dilute base (6.25 mM NaOH), 10X base (62.5 mM NaOH), or dilute acid (4% v/v H_2_SO_4_). Free glucose amounts were quantified and subtracted from glucose values following the digestions involving grinding alone or grinding plus hot water or acid in order to determine the amounts of sugars arising solely from cell wall polysaccharides. This subtraction was not necessary for the digestions involving base as those pretreatments degrade any free glucose present before neutralization and hydrolytic enzyme treatment.

Significant differences in digestibility were observed among the seven inbred lines (**Table 3**). For example, glucose saccharification amounts following the grinding-alone pretreatment ranged from a low of 4.8% yield dry weight (dw) to a high of 5.9% yield dw, a 21% difference; Pentose saccharification amounts for this same pretreatment differed by as much as 20% (**Table 3**). Performing a very mild alkaline pretreatment (6.25 mM NaOH; dilute base) before enzymatic saccharification (**Table 3**) more than doubled glucose and pentose yields compared to the yields following the grinding-alone pretreatment (**Table 3**); differences between the lines pretreated with dilute base were as much as 38% for glucose and 44% for pentoses.

**Table 3:** *Brachypodium distachyon* biomass digestibilities associated with a panel of pretreatments followed by enzymatic hydrolysis.

Line	Sugar	Grinding alone	Grinding plus:			
			Hot water	6.25 mM NaOH	62.5 mM NaOH	4% H_2_SO_4_
Bd21	Glucose	5.5 ± 0.2^ab^	5.8 ± 0.1^a^	14.8 ± 0.3^a^	23.4 ± 0.4^ab^	7.6 ± 0.3^a^
Bd21-3		5.4 ± 0.1^ab^	5.4 ± 0.2^ab^	12.0 ± 0.2^b^	22.8 ± 0.3^abc^	6.6 ± 0.3^ab^
Bd30-1		5.1 ± 0.2^ab^	5.5 ± 0.2^ab^	13.1 ± 0.4^b^	23.4 ± 0.4^ab^	5.9 ± 0.3^bc^
Bd3-1		4.9 ± 0.2^b^	5.4 ± 0.2^ab^	12.1 ± 0.4^b^	21.3 ± 0.6^c^	5.2 ± 0.2^c^
Bd2-3		5.1 ± 0.3^ab^	6.0 ± 0.2^a^	10.3 ± 0.3^c^	21.8 ± 0.5^bc^	5.7 ± 0.7^bc^
Bd1-1		5.9 ± 0.2^a^	5.6 ± 0.4^a^	13.3 ± 0.4^b^	24.9 ± 0.3^a^	6.1 ± 0.4^bc^
Bd18-1		4.8 ± 0.2^b^	4.6 ± 0.2^b^	10.1 ± 0.3^c^	22.1 ± 0.7^bc^	5.9 ± 0.3^bc^
*F* ratio		3.44	4.62	26.83	6.35	6.56
Prob.		0.0047	0.0005	<0.0001	<0.0001	<0.0001

Bd21	Pentoses	1.1 ± 0.04^b^	1.1 ± 0.04^bc^	6.1 ± 0.1^a^	9.7 ± 0.2^bc^	9.0 ± 0.2^b^
Bd21-3		1.1 ± 0.04^bc^	1.1 ± 0.04^bc^	5.2 ± 0.1^bc^	10.9 ± 0.1^a^	9.3 ± 0.2^b^
Bd30-1		1.0 ± 0.04^c^	1.0 ± 0.06^c^	5.7 ± 0.2^ab^	10.5 ± 0.3^ab^	9.6 ± 0.2^ab^
Bd3-1		1.0 ± 0.04^bc^	1.1 ± 0.05^bc^	4.9 ± 0.2^c^	9.3 ± 0.3^c^	9.2 ± 0.2^b^
Bd2-3		0.9 ± 0.03^c^	1.2 ± 0.03^b^	3.9 ± 0.2^d^	10.3 ± 0.2^ab^	9.4 ± 0.3^ab^
Bd1-1		1.3 ± 0.05^a^	1.6 ± 0.08^a^	5.8 ± 0.2^ab^	11.1 ± 0.2^a^	10.4 ± 0.3^a^
Bd18-1		1.0 ± 0.04^bc^	1.2 ± 0.04^b^	4.0 ± 0.1^d^	10.7 ± 0.3^ab^	10.0 ± 0.3^ab^
*F* ratio		11.88	16.07	31.93	7.85	3.90
Prob.		<0.0001	<0.0001	<0.0001	<0.0001	0.0020

*Values represent glucose and pentose sugars yield (% dry weight) means ± SEM. Free glucose amounts were subtracted from glucose means for grinding alone, and grinding plus either hot water or dilute acid pretreatments. Different letters indicate significant differences; α′ = 0.003.*

Relative saccharification efficiencies for the various lines differed when considering the different pretreatments (**Table 3**). In other words, the line that released the most glucose with grinding alone (Bd1-1) was not the same line that released the most glucose with dilute acid pretreatment (Bd21). Moreover, the line that released the most glucose with a given pretreatment was not always the same line that released the most pentoses.

### Phenotypic Correlations

The correlations between growth habit and cell wall composition variation among the seven inbred lines is shown in **Table 4**. Aboveground biomass weights were found to be strongly positively correlated with the amounts of cell wall polysaccharide-derived sugars arabinose (*r* = 0.59, *p* < 0.0001), xylose (*r* = 0.65, *p* < 0.0001), and galactose (*r* = 0.48, *p* < 0.0001). Conversely, aboveground biomass weights were negatively correlated with S (*r* = -0.76, *p* < 0.0001) and H (*r* = -0.40, *p* = 0.0002) lignin unit amounts, as well as with crystalline cellulose (*r* = -0.49, *p* < 0.0001). ABSL and G lignin unit amounts were both strongly correlated with plant height (ABSL, *r* = 0.61, *p* < 0.0001; G, *r* = 0.64, *p* < 0.0001) and with each other (*r* = 0.71, *p* < 0.0001), but neither were correlated with either mass or HD. Lignin S unit amounts were strongly correlated with H unit amounts (*r* = 0.58, *p* < 0.0001) and strongly negatively correlated with xylose (*r* = -0.69, *p* < 0.0001), arabinose (*r* = -0.64, *p* < 0.0001), and galactose (*r* = -0.55, *p* < 0.0001). HD, which had a very strong negative correlation with lignin S unit amounts (*r* = -0.90, *p* < 0.0001), showed strong positive correlations with both weight (*r* = 0.73, *p* < 0.0001) and culm number (*r* = 0.65, *p* < 0.0001), and also had corresponding positive correlations with xylose (*r* = 0.61, *p* < 0.0001), arabinose (*r* = 0.56, *p* < 0.0001), and galactose (*r* = 0.53, *p* < 0.0001). Xylose, arabinose, and galactose, were not only very strongly positively correlated with each other (*r* = 0.58 to 0.90, *p* < 0.0001), but also were very strongly correlated to the number of culms (*r* = 0.63 to 0.72, *p* < 0.0001).

**Table 4:** Correlation coefficients between growth traits and cell wall components.

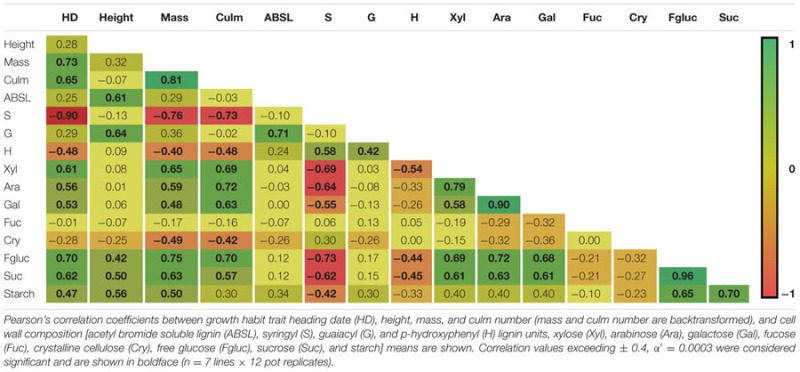

**FIGURE 4:**
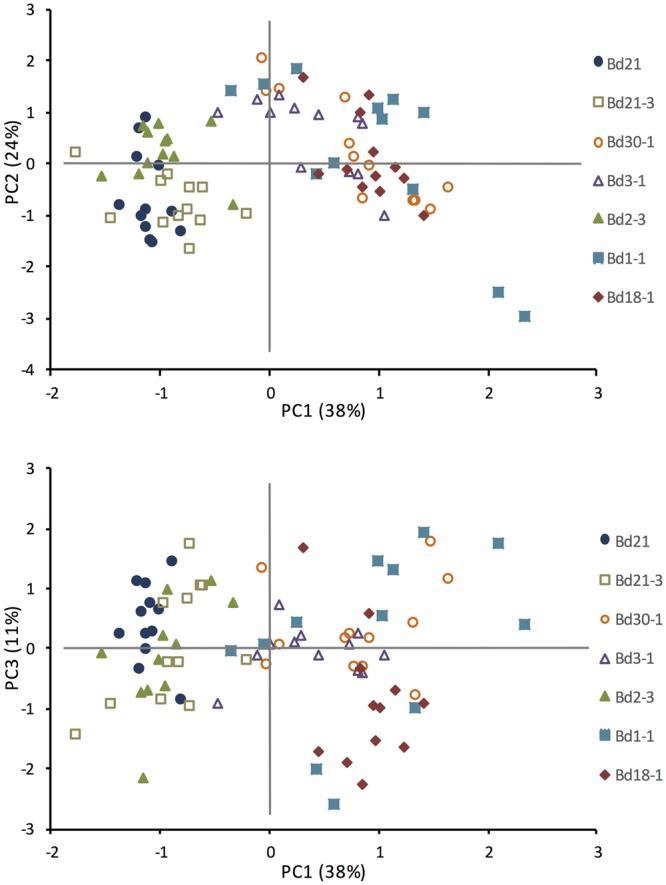
**Principal component analysis (PCA) based upon phenotypic growth and cell wall composition observations.** Means included in the PCA were HD, biomass index (BI = height × mass/culm number), and amounts for ABSL, lignin S, G, and H units (from thioacidolysis), xylose, arabinose, galactose, fucose, and crystalline cellulose. *N* = 84, 7 lines × 12 biological (pot) replicates. Rotated factor pattern and final communality estimates are in Supplementary Table S1. Significant principal components (PC) are in Supplementary Table S2.

PC analysis was performed to reduce the number of variables that described the observed differences in growth trait and cell wall composition of the inbred lines. The first three PC displayed eigenvalues greater than one, which was supported by a scree test. The three PCs that explained 73% of the total variation are shown in two-dimensional scatter plots (**Figure 4**). The first principal component (PC1) accounted for 38% of the observed variation, and clustered Bd21, Bd21-3, and Bd2-3 together and Bd1-1, Bd18-1, and Bd30-1 together based upon positive factor loadings HD, and amounts of xylose, arabinose, and galactose, and inverse factor loading from lignin S and H unit amounts. PC2 accounted for 24% of the observed variation with factor loadings of BI, total lignin (ABSL) and lignin G unit amounts resulting in slight separations of Bd2-3 from Bd21, and Bd3-1 and Bd1-1 from Bd30-1 and Bd18-1. PC3 accounted for an additional 11% of the observed variation with positive factor loading of crystalline cellulose amount, and inverse factor loading from xylose and arabinose, that slightly separated Bd1-1 from Bd3-1. The rotated factor loadings and the PC are shown in Supplementary Tables S1 and S2.

To ascertain whether any of the variation in growth characteristics or cell wall composition might correlate with differences in biomass digestibility, Pearson’s correlation coefficients were used to correlate the three significant PC with the sugar release data from our five digestibility treatments. A significant negative correlation was observed between PC1 with glucose release following grinding plus 4% (v/v) H_2_SO_4_ pretreatment, and PC3 with glucose release following grinding plus 6.2 mM NaOH pretreatment (**Table 5**).

**Table 5:** Correlation coefficients between principal components (PC) and biomass digestibility following five pretreatments.

	Pretreatment									
Principal component		Grinding alone		Grinding plus hot water		Grinding plus 6.2 mM NaOH		Grinding plus 62 mM NaOH		Grinding plus 4% (v/v) H	
	Gluc	Pent	Gluc	Pent	Gluc	Pent	Gluc	Pent	Gluc	Pent
PC1	-0.14	-0.01	-0.08	0.21	-0.10	-0.02	0.09	0.14	-0.41*	0.20
PC2	-0.03	0.05	-0.08	0.11	0.08	0.04	-0.10	-0.16	-0.13	-0.09
PC3	0.22	0.16	0.14	0.06	0.36*	0.31	0.11	-0.10	0.18	-0.09

*Principal components derived from heading date, biomass index, and cell wall composition correlated with enzymic saccharification of glucose (Gluc) and pentoses (Pent) following five thermochemical pretreatments (*n* = 7 lines × 12 biological replicates). ^∗^Correlation values exceeding ± 0.35.*

### Effects of Vernalization Treatments on Biomass Traits

**FIGURE 5:**
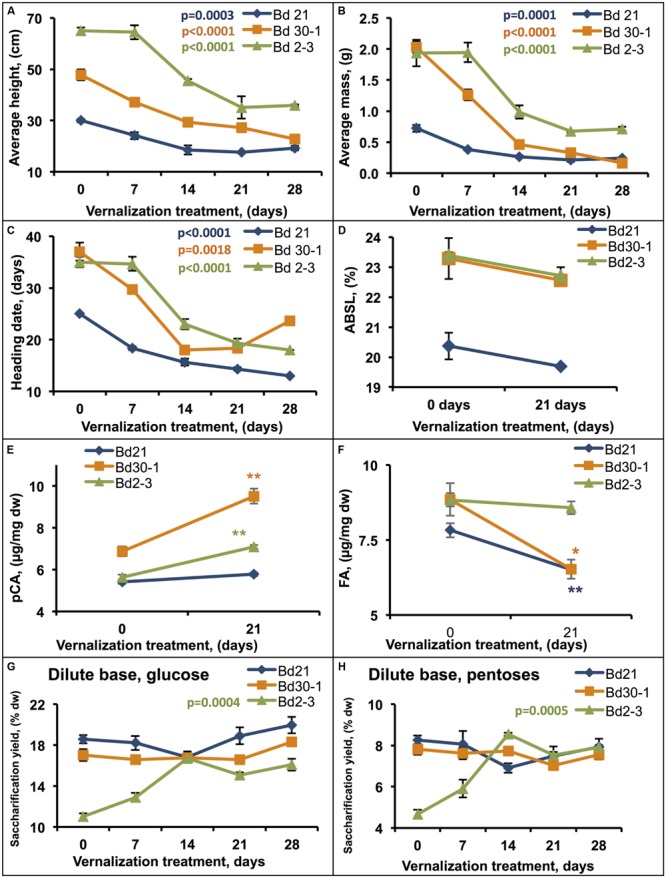
**Effects of vernalization treatment duration on biomass traits.** Plants that were vernalized for either 0, 7, 14, 21, or 28 days, transferred to a growth chamber, and then grown to maturity were assessed for **(A)** height, **(B)** aboveground dry mass, and **(C)** heading date. Stem plus leaf sheath biomass was assessed for **(D)** ABSL, **(E)** *p*-coumaric acid (*p*CA), and **(F)** ferulic acid (FA) content, and hydrolytic enzyme digestibility following dilute base (6.25 mM NaOH) pretreatment [**(G)** glucose, **(H)** pentoses]. Regression was performed to determine the effect vernalization duration on **(A–C,G,H)**, α′ = 0.002. Significant differences indicated for **(E,F)** were determined by Student’s *T*-test (^∗^ = 0.05 > ρ > 0.01; ^∗∗^ = 0.01 > ρ > 0.0005). % dw, percent dry weight. Bars indicate SEM. *N* = 9, *n* = 3 where *n* is the number of pots for each line (each pot contained four plants that were measured together to generate a sample mean).

To investigate the effect that vernalization had on biomass traits, three spring habit inbred lines (Bd21, Bd30-1, and Bd2-3) exhibiting a range of phenotypic differences under standard growth conditions were subjected to vernalization treatments ranging from 1 week (7 days) to 4 weeks (28 days) at 6°C. Increasing vernalization duration was highly correlated with reduced average height, aboveground mass, and HD for all three lines (**Figures 5A–C**). Bd21, the relatively shortest inbred line, had a large 50% reduction in height in response to vernalization that was correlated to vernalization duration (*b*_1_ = -0.40, adjusted *r*^2^ = 0.63). Similarly, Bd30-1 showed a significant 56% reduction in height after 21 days of vernalization, with an overall height and vernalization duration correlation (*b*_1_ = -0.86, adjusted *r*^2^ = 0.89). Bd2-3, the tallest accession, had a 60% reduction in height after 21 days of vernalization treatment, and a strong correlation between height and duration of vernalization (*b*_1_ = -1.25, adjusted *r*^2^ = 0.81). After 21 days of vernalization, Bd21, Bd30-1, and Bd2-3 also showed significant aboveground mass reductions of 71, 85, and 63%, respectively, that were correlated to vernalization duration (Bd21, *b*_1_ = -0.02, adjusted *r*^2^ = 0.66; Bd30-1, *b*_1_ = -0.07, adjusted *r*^2^ = 0.86 and Bd2-3, *b*_1_ = -0.05, adjusted *r*^2^ = 0.75; **Figure 5B**). Increasing the length of vernalization treatment also significantly reduced the HD (43 to 48% reductions; Bd21, *b*_1_ = -0.40, adjusted *r*^2^ = 0.84; Bd30-1, *b*_1_ = -0.54, adjusted *r*^2^ = 0.50 and Bd2-3, *b*_1_ = -0.71, adjusted *r*^2^ = 0.85; **Figure 5C**).

Measurements of ABSL lignin in senesced Bd21, Bd30-1, and Bd2-3 stems plus leaf sheaths from the vernalized plants after maturation identified no significant differences across vernalization lengths, although all three lines showed slight reductions (**Figure 5D**). On the other hand, the amounts of cell wall hydroxycinnamic acids released upon 2 M NaOH treatment did change significantly in response to vernalization (**Figures 5E,F**). *p*CA amounts increased by 32 and 24% for Bd30-1 and Bd2-3, respectively, whereas FA amounts decreased by 30 and 18% for Bd30-1 and Bd21, respectively.

To assess the effect of vernalization on biomass digestibility, we employed a digestion assay using a dilute base pretreatment. For Bd21 and Bd30-1, the amounts of glucose and pentose released varied little with increased vernalization durations (**Figures 5G,H**). In contrast, 14 days or longer vernalization of Bd2-3 resulted in as much as 41 and 59% increases in glucose and pentoses yields, respectively, compared to that released from unvernalized stem biomass (**Figures 5G,H**). The increased digestibility results correlated strongly with the vernalization duration (glucose, *b*_1_ = 0.18, adjusted *r*^2^ = 0.61; pentose, *b*_1_ = 0.12, adjusted *r*^2^ = 0.59).

**FIGURE 6:**
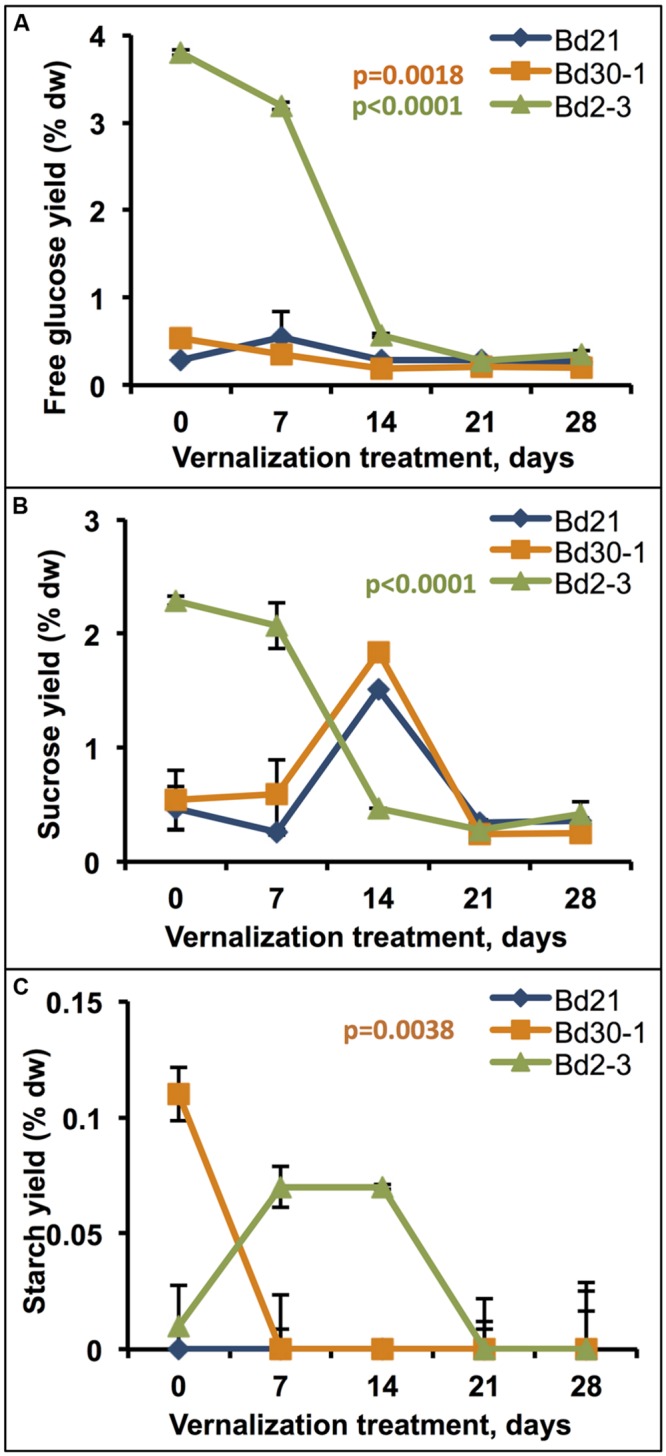
**Effects of vernalization treatment duration on free glucose, sucrose, and starch content of senesced biomass.** Stem plus leaf sheath biomass from plants that were vernalized for either 0, 7, 14, 21, or 28 days, transferred to a growth chamber, and then grown to maturity was assessed for **(A)** free glucose, **(B)** sucrose, and **(C)** starch content. Regression was performed to determine the effect of increasing vernalization treatment for each compound quantitated, α′ = 0.02. % dw, percent dry weight. Bars indicate SEM. *N* = 9, *n* = 3.

Vernalization also had dramatic effects on senesced stem plus leaf sheath free glucose, sucrose, and starch levels. Twenty-one days of vernalization decreased Bd2-3 free glucose (*b*_1_ = -0.14, adjusted *r*^2^ = 0.81) and sucrose (*b*_1_ = -0.08, adjusted *r*^2^ = 0.74) levels, reducing those sugar levels to the same observed in Bd30-1 and Bd2-3 with or without vernalization (**Figures 6A,B**). Bd30-1 starch content, which was relatively higher in Bd30-1 senesced stems plus leaf sheaths compared to Bd21 and Bd2-3, dropped to near-zero after only 7 days of vernalization (*b*_1_ = -0.003, adjusted *r*^2^ = 0.45; **Figure 6C**).

## Discussion

Grass vegetative biomass holds considerable potential as a feedstock for the generation of liquid biofuels owing to its high latent sugar content of approximately two thirds polysaccharides by dw (Pauly and Keegstra, 2008) and widespread availability, with hundreds of millions of tons available annually in the U.S. alone (U.S. Department of Energy, 2011). *B. distachyon* (*Brachypodium*) has emerged as a tractable model for studying a variety of traits (Brkljacic et al., 2011). In this study, we quantified and compared cell wall composition differences between a set of seven *Brachypodium* inbred lines previously found to have a high level of genotypic diversity. We found considerable phenotypic variation, suggesting that studies of related RILs can identify underlying genetic pathways differences, the knowledge of which can be used to improve grasses for use in generating biofuels. This study builds upon work by Tyler et al. (2014), who semi-quantitatively assessed polysaccharides composition but not lignin or biomass recalcitrance of a collection of *Brachypodium* lines.

Our analyses identified strong positive correlations between ABSL lignin amounts, lignin G unit amounts and plant heights (**Table 4**). It may be that taller stems accumulate more cell-wall-strengthening lignin richer in G units to facilitate crosslinking in response to greater bending and torsional stresses. In addition, higher solute pressures in xylem and phloem may be associated with the taller stems, which may induce lignin accumulation to strengthen the vasculature.

Dramatic differences between the inbred lines were also identified with respect to lignin S unit content and S:G ratios (**Figures 3A–C**). Studies by others of *Brachypodium* and *Arabidopsis* enhanced saccharification mutants identified correlations between reduced S:G ratios and improved biomass digestibility (Van Acker et al., 2013; Marriott et al., 2014; Silveira et al., 2015). However, our statistical analyses identified only a few correlations between lignin composition and biomass digestibility when considering all five digestibility treatments employed (**Table 5**; Supplementary Table S1; **Figure 4**). One possible reason why there was not a clearer observed relationship between lignin and digestibility is that it may have been masked by significant effects of other cell wall components such as hemicelluloses, AGPs, arabinogalactans (AGs), extensins, and/or pectins. Consistent with this hypothesis, a study of a maize RIL population identified several genetic determinants for digestibility, none of which were associated with lignin (Penning et al., 2014). Taken together, these results suggest that studies of the appropriate *Brachypodium* RILs could identify not only allelic variants controlling flux through the monolignol biosynthetic pathway but also determinants unrelated to lignin that underlie the observed biomass digestibility differences.

Significant phenotypic variation was also observed in the quantified total amounts of neutral sugars derived from cell wall polysaccharides and other sugar-containing wall components (**Table 1**). For example, Bd18-1 had 16% more xylose and 25% more arabinose in senesced stems plus leaf sheaths than Bd21. Most of the xylose and a portion of the arabinose in *Brachypodium* and other grasses is present in the hemicellulose glucuronoarabinoxylan (GAX; Carpita and Gibeaut, 1993; McCann and Carpita, 2008; Vogel, 2008; Scheller and Ulvskov, 2010). Thus it seems likely that these inbred lines vary in their relative amounts of GAX. Arabinose is also present in AGPs, AGs, extensins, and pectins (Carpita, 1996; Seifert and Roberts, 2007). The relative amounts of these cell wall components in *Brachypodium* remain to be quantitatively determined, although Tyler et al. (2014) did detect relatively stronger signals with the AGP-specific JIM13 monoclonal antibody, compared to various pectin-specific antibodies, in Brachypodium CDTA- and NaOH-extracted cell wall materials.

Our results also revealed that the amounts of galactose and rhamnose, which are predominantly found in pectins and AGPs (Carpita, 1996; Seifert and Roberts, 2007; Caffall and Mohnen, 2009), varied significantly between inbred lines (**Table 1**). Tyler et al. (2014) identified similar significant differences in pectin composition between *Brachypodium* inbred lines by using a CoMPP technique, employing five antibodies to detect epitopes of the pectin homogalacturonan (HG). Their study identified Bd21-3 and Bd21 as having the largest and smallest relative amounts of HG, with Bd3-1, Bd30-1, and Bd1-1 falling in between. Those relative differences in HG levels matched well with the relative differences in galactose amounts our analyses uncovered between the same inbred lines (**Table 1**), thus providing cross-validation between the two studies.

Our results showed that galactose levels (likely from pectins and AGPs) along with xylose and arabinose levels (xylose predominantly from hemicelluloses, and arabinose mostly from hemicelluloses, AGPs, extensins, and pectins) were strongly and positively correlated with biomass accumulation (**Table 4**). Considerable evidence in dicotyledonous plants points toward pectin composition and structure, including differences in pectin methylesterification, influencing plant morphogenesis and growth (Palin and Geitmann, 2012; Kim et al., 2015). AGPs also play essential roles in growth and development (Tan et al., 2012). Pectins and AGPs have been studied relatively little in monocots, including grasses (Xiao and Anderson, 2013). Therefore, these *Brachypodium* inbred lines could be useful tools for related studies.

Besides the aforementioned differences in structural polysaccharide levels between the inbred lines, substantial differences in free glucose, sucrose, and starch amounts were identified (**Table 2**). For example, free glucose amounts varied by as much as 10.75-fold in senesced stem plus leaf sheath tissues. Intriguingly, the relatively high glucose and sucrose amounts in Bd2-3 senesced stems plus leaf sheaths largely disappeared when plants were vernalized for 14 days, dropping to the amounts found in unvernalized Bd21 and Bd30-1 (**Figure 6**). Taken together, these findings suggest there are likely genetic differences in how these lines partition and store carbohydrates. Slewinski (2012) pointed out the importance of understanding carbon sink-source dynamics in order to maximize grass crop yields and improve yield stability under stress conditions. Studies employing *Brachypodium* could be informative in this regard.

## Conclusion

This study identified considerable phenotypic diversity for a variety of biomass-related traits in a genotypically diverse set of seven *Brachypodium* inbred lines. The phenotypic differences between lines for a given trait are significantly large enough to warrant follow-up genetic studies of related RILs in order to identify the underlying genetic determinants. For example, a Bd21 × Bd3-1 RIL population was successfully employed to fine-map a Barley Stripe Mosaic Virus resistance gene (e.g., Cui et al., 2012). We have analyzed biomass digestibility in a Bd21 × Bd2-3 RIL population; our preliminary findings suggest that a few genetic loci underlie the majority of observed digestibility differences and that transgressive segregation may be occurring.

Although multivariate analysis of the data from this study identified correlations between growth characteristics, cell wall compositions, and biomass digestibility, further analyses will be required to identify cause and effect relationships. Moreover, given that the number of lines analyzed were limited, it may be worth revisiting some of these traits using a larger number of lines so as to increase statistical power. This may uncover even larger phenotypic differences as well as confirm or rule out marginal or unexpected correlations.

## Author Contributions

CC, JR, DG, and JS conceived of the experiments and interpreted data. CC, AL, NS, CF, SK, and RS carried out the experiments and analyzed data. CC, DG, and JS wrote the manuscript. All authors edited and approved the final manuscript.

## Conflict of Interest Statement

The authors declare that the research was conducted in the absence of any commercial or financial relationships that could be construed as a potential conflict of interest.
