# Supplementary material for: Cell Wall Composition and Biomass Recalcitrance Differences Within a Genotypically Diverse Set of Brachypodium distachyon Inbred Lines
Source: Front Plant Sci. 2016 May 26;7:708. doi: 10.3389/fpls.2016.00708 (PMC4880586; doi:10.3389/fpls.2016.00708)
Supplement: Supplementary file 1 [file Table_1.PDF]

SUPPLEMENTAL DATA

Cell wall composition and biomass recalcitrance differences within a genotypically diverse set of *Brachypodium distachyon* inbred lines

Cynthia L. Cass,[1,2] Anastasiya A. Lavell,[3] Nicholas Santoro,[4] Cliff E. Foster,[4] Steven D. Karlen,[2] Rebecca A. Smith,[2] John Ralph,[2,5] David F. Garvin,[3,6] and John C. Sedbrook[1,2*]

[1]School of Biological Sciences, Illinois State University, Normal, IL 61790, USA

[2]U.S. Department of Energy Great Lakes Bioenergy Research Center, University of Wisconsin, Madison, WI 53726, USA

[3]Department of Agronomy and Plant Genetics, University of Minnesota, St. Paul, MN 55108, USA

[4]U.S. Department of Energy Great Lakes Bioenergy Research Center, Michigan State University, East Lansing, MI 48824, USA

[5]Department of Biochemistry, Wisconsin Energy Institute, University of Wisconsin, Madison, WI, 53706, USA

[6]USDA Agricultural Research Service, Plant Science Research Unit, St. Paul, MN 55108, USA

*Address for correspondence: John Sedbrook, School of Biological Sciences, Illinois State University, Normal, IL 61790, phone 309-438-3374, fax 309-438-3722, email jcsedbr@ilstu.edu

Supplemental Table 1. Rotated factor pattern and final communality estimates (h^2) of the principal component analysis (PCA) of growth trait and cell wall phenotypic means.

	PC1		PC2		PC3		h^2
**HD**	85	*	31		-22		0.89
**BI**	14		85	*	12		0.75
**ABSL**	-2		86	*	-13		0.76
**S**	-90	*	-13		23		0.88
**G**	-6		90	*	-17		0.87
**H**	-81	*	28		-25		0.81
**Xyl**	80	*	0		-21		0.76
**Ara**	63	*	-14		-58	*	0.91
**Gal**	52	*	-14		-63	*	0.87
**Fuc**	-3		-2		-3		0.88
**Cry**	-9		-22		80	*	0.71

Heading date (HD), biomass index (BI = height x mass / culm number), and amounts for acetyl bromide soluble lignin (ABSL), Syringyl (S), Guaiacyl (G), and *p*-Hydroxyphenyl (H) lignin units, xylose (Xyl), arabinose (Ara), galactose (Gal), fucose (Fuc), and crystalline cellulose (Cry). N = 84, n = 12 biological (pot) replicates for each of seven lines, each pot contained five plants each, $p < 0.05$.

Supplemental Table 2. Principal components used to determine the correlation of growth trait and cell wall phenotypes with digestibility.

Line	Obs	PC1	PC2	PC3
Bd21	1	-1.08374	-1.52166	0.30979
Bd21	2	-1.15199	-1.24545	1.09495
Bd21	3	-1.1501	-0.90315	0.28314
Bd21	4	-1.18328	-1.00937	0.63463
Bd21	5	-1.10341	-1.48864	0.77703
Bd21	6	-0.82059	-1.32833	-0.84606
Bd21	7	-0.902	-0.93098	1.47794
Bd21	8	-1.02082	-0.02692	0.65015
Bd21	9	-1.22085	0.12113	1.13951
Bd21	10	-1.13793	0.87733	0.0133
Bd21	11	-1.21217	0.68204	-0.31606
Bd21	12	-1.38663	-0.789	0.278
Bd21_3	13	-0.8454	-1.00748	-0.19997
Bd21_3	14	-0.75533	-0.87954	0.86121
Bd21_3	15	-1.46309	-1.07903	-0.88792
Bd21_3	16	-0.98327	-1.16469	0.78388
Bd21_3	17	-0.6295	-0.48606	1.05682
Bd21_3	18	-0.74508	-1.65055	1.77057
Bd21_3	19	-0.64474	-1.08655	1.07873
Bd21_3	20	-0.2161	-0.99006	-0.18728
Bd21_3	21	-0.73801	-0.45337	-0.93862
Bd21_3	22	-1.0094	-0.34287	-0.82708
Bd21_3	23	-0.93589	-0.21598	-0.21557
Bd21_3	24	-1.78878	0.21953	-1.41459
Bd2_3	25	-1.19533	-0.20937	-0.70724
Bd2_3	26	-0.3344	-0.82669	0.76369
Bd2_3	27	-1.54714	-0.25369	-0.05773
Bd2_3	28	-0.98853	0.17071	0.21737
Bd2_3	29	-1.1257	0.01743	-0.6895
Bd2_3	30	-0.94169	0.46936	1.00985
Bd2_3	31	-0.9721	0.42466	-0.6028
Bd2_3	32	-0.87395	0.1186	0.09342
Bd2_3	33	-1.03105	0.7882	-0.17074
Bd2_3	34	-1.17308	0.72671	-2.14016
Bd2_3	35	-1.12234	0.58876	-0.67173
Bd2_3	36	-0.54423	0.81433	1.15535
Bd30_1	37	1.47191	-0.87405	1.78343
Bd30_1	38	0.83716	-0.67627	-0.29844

Bd30_1	39	1.31117	-0.72018	0.45596
Bd30_1	40	1.32481	-0.73067	-0.7696
Bd30_1	41	0.89325	-0.05311	0.19115
Bd30_1	42	0.0753	1.46523	0.09319
Bd30_1	43	-0.03768	1.40279	-0.23099
Bd30_1	44	0.71633	0.38354	0.26831
Bd30_1	45	-0.07903	2.0509	1.3772
Bd30_1	46	0.68092	1.29576	0.18356
Bd30_1	47	1.62241	-0.45979	1.16247
Bd30_1	48	0.76468	0.13869	-0.27148
Bd3_1	49	0.72885	-0.1684	0.07915
Bd3_1	50	1.04459	-1.0055	-0.11303
Bd3_1	51	0.79468	-0.22086	-0.35116
Bd3_1	52	0.28587	-0.09861	0.2208
Bd3_1	53	-0.11394	1.23047	-0.09722
Bd3_1	54	-0.49114	0.99117	-0.89256
Bd3_1	55	0.83694	0.75786	-0.40821
Bd3_1	56	-0.00844	0.98446	0.0825
Bd3_1	57	0.79454	0.90038	0.24865
Bd3_1	58	0.45062	0.93501	-0.088
Bd3_1	59	0.07419	1.32043	0.75018
Bd3_1	60	0.21311	1.08431	0.13787
Bd1_1	61	0.97895	1.07239	1.47562
Bd1_1	62	1.41081	0.99628	1.93924
Bd1_1	63	1.03113	0.84261	0.54952
Bd1_1	64	0.24329	1.84857	0.43126
Bd1_1	65	2.32863	-2.96547	0.42332
Bd1_1	66	2.09376	-2.52227	1.75116
Bd1_1	67	0.57572	0.0051	-2.56989
Bd1_1	68	0.41152	-0.20039	-1.98141
Bd1_1	69	1.31412	-0.5143	-0.96405
Bd1_1	70	-0.36623	1.40932	-0.03504
Bd1_1	71	-0.06564	1.51759	0.07951
Bd1_1	72	1.13105	1.2456	1.31569
Bd18_1	73	0.81746	1.0005	-0.32585
Bd18_1	74	0.91125	1.3219	0.59274
Bd18_1	75	1.01056	-0.57087	-0.95828
Bd18_1	76	0.83388	-0.46644	-2.25763
Bd18_1	77	0.94489	0.21978	-0.95378
Bd18_1	78	0.69977	-0.11768	-1.88725
Bd18_1	79	0.45047	-0.21642	-1.68661
Bd18_1	80	0.96899	-0.26287	-1.50746
Bd18_1	81	1.40152	-1.00507	-0.91782

Bd18_1	82	0.29607	1.6727	1.6989
Bd18_1	83	1.13495	-0.0866	-0.67761
Bd18_1	84	1.22962	-0.28686	-1.62429

Principal components summarizing the observed variation across and among means for heading date (HD), biomass index (BI, height x mass / culm number), and amounts of acetyl bromide soluble lignin (ABSL), S, G, and H lignin monomers (from thioacidolysis), xylose, arabinose, galactose, fucose, and crystalline cellulose. Obs = observation number, N = 84, n = 12 biological (pot) replicates, density = five plants per pot.

$p < 0.05$).
